# Function of *Vitellogenin receptor* gene in reproductive regulation of *Zeugodacus cucurbitae* (Coquillett) after short-term high-temperature treatment

**DOI:** 10.3389/fphys.2022.995004

**Published:** 2022-10-04

**Authors:** Yuyang Lian, Sihua Peng, Jingjing Jia, Jinlei Li, Aqiang Wang, Shuyan Yang, Rongjiao Zheng, Xiaofeng Yang, Shihao Zhou

**Affiliations:** ^1^ Sanya Nanfan Research Institute of Hainan University, Sanya, China; ^2^ Key Laboratory of Germplasm Resources Biology of Tropical Special Ornamental Plants of Hainan Province, College of Forestry, Hainan University, Haikou, China; ^3^ Key Laboratory of Plant Disease and Pest Control of Hainan Province, Haikou, China

**Keywords:** short-time high temperature, *Vitellogenin receptor* gene, *Zeugodacus cucurbitae* (Coquillett), oviposition, reproduction

## Abstract

Climate change has increased the frequency of extreme heat events. *Zeugodacus cucurbitae* (Coquillett) is an important tropical pest that typically changes its reproductive strategies in response to extremely high temperatures. Newly emerged adults of three consecutive generations (F_1_, F_2_, and F_3_) of *Z. cucurbitae* (Coquillett) were exposed to 25°C, 33°C, 37°C, 41°C, and 45°C treatments for 1 h to clarify the effects of short-term high temperatures on its reproduction. The influence of these temperatures on reproduction was evaluated using indicators, such as egg number. Newly emerged adults were exposed to 25°C and 45°C treatments for 1 h, and the expression of *Vitellogenin receptor* (*VgR*) gene in females was interfered with siRNA, and silencing efficiency of RNAi was evaluated. Results showed that short-term high temperatures, except for F_1_ treated at 45°C for 1 h to stimulate oviposition, exert a general adverse effect on the reproduction of *Z. cucurbitae* (Coquillett). All F_3_ died after the 45°C treatment for 1 h. Silencing of the *VgR* gene resulted in the significant downregulation of *VgR* gene expression at both 24 and 72 h. The egg number, oviposition days, and hatchability of eggs were significantly lower than those of other treatment groups after interference, and the inhibition effect of egg number was the most evident, with a decrease of 88.4% and 95.2% at 25°C and 45°C, respectively, compared with that of the Control Check (CK). Ovarian development speed and diameter were also significantly lower than those of other treatment groups after the interference. The results of this study can provide a theoretical reference for the integrated control of *Z. cucurbitae* (Coquillett) during high-temperature seasons.

## 1 Introduction

Global warming has become a key issue in global climate change. Species have begun to change their reproductive strategies to improve their reproduction with the increase of temperatures (Bale et al., 2002; Pedl et al., 2017). Insects are typical poikilotherms with small body sizes, thin body walls, and poor self-thermal regulation ability, thereby increasing the frequency of their thermal exchanges with the environment (Yamamura et al., 2006). The majority of insects present multiple generations every year and typically adapt to temperature changes in a short period of time (Raza et al., 2015). Meanwhile, the adaptation of pests to high temperatures will cause large economic losses to humans. Investigating the effect of high temperatures on pest reproduction can help us formulate scientific pest control measures. Lv et al. (2021) revealed that rearing *Cnaphalocrocis medinalis* at 34°C results in remarkably lower fecundity than those at 18°C and 22°C. Rearing of *Aenasius bambawalei* at 36°C results in fast gonad development, increased egg and sperm production, and enhanced reproduction capacity (Zhang et al., 2021). However, the frequency and intensity of extreme heat events are increasing (Ma and Ma, 2016), insects experience short and extreme heat rather than constant heat, and extreme heat may drive insect responses to climate change more than average temperature in the context of global warming (Ma et al., 2021).

*Zeugodacus cucurbitae* (Coquillett) (Diptera: Tephritidae) is native to India and now found in most tropical and subtropical regions; *Z. cucurbitae* (Coquillett) can parasitize more than 120 species of fruits and vegetables as well as lay eggs in fruits and vegetables until they hatch and feed inside the fruit, thereby causing the fruit to rot (He et al., 2019). Compared with other fruit flies, *Z. cucurbitae* (Coquillett) is more harmful to farmland because it can also parasitize flowers and stems, even in root tissues, aside from fruits and vegetables. Female adults lay eggs in unopened female and male flowers, and larvae can successfully develop in primary roots, stems, and petioles (Back and Pemberton, 1914). Understanding the effects of short-term high temperatures on the reproduction of *Z. cucurbitae* (Coquillett) can help formulate scientific control strategies for *Z. cucurbitae* (Coquillett) in high-temperature seasons.

The optimum temperature for *Z. cucurbitae* (Coquillett) is 25°C–30°C (Wei et al., 2011). High temperature is the condition wherein the external temperature is higher than the upper limit of the optimum temperature (>30°C) (Zeng et al., 2018). Zeng et al. (2019) applied temperatures of 25°C (control group), 33°C, 37°C, 41°C, and 45°C to treat the contemporary (F_1_) adult *Z. cucurbitae* (Coquillett) for 1 h and recorded indicators, such as egg number, to clarify the apparent biological effects of short-term high temperature on F_1_ reproduction. Zhou et al. (2020) explored transcriptional and protein level expression based on high-throughput sequencing technology and revealed that the *Vitellogenin receptor* (*VgR*) gene is differentially expressed at both transcriptional and protein levels at the molecular biology level after *Z. cucurbitae* (Coquillett) is exposed to short-term high temperatures of 25°C, 33°C, 37°C, 41°C, and 45°C for 1 h. VgR is a protein necessary for the entry of Vitellogenin (Vg) into the developing oocytes (Yang et al., 2020); a receptor that can mediate endocytosis, usually with ovarian specificity, and a specific endocytosis receptor for Vg (Tufail and Takeda, 2005). The majority of insects express *VgR* genes in large amounts before mating or egg laying, and various female insects present different maturation stages of ovaries and trends in *VgR* gene expression (Tufail and Takeda, 2009). Exploring the expression dynamics of the *VgR* gene in pests can provide a powerful theoretical basis for pest control (Wang et al., 2016). VgR on the surface of the oocyte decreased and transporting Vg to the oocyte was impossible when the *VgR* gene of *Blattella germanica* was silenced, thereby resulting in the large accumulation of Vg in the fat body and stagnant development of the oocyte (Ciudad et al., 2006). Interference with the *VgR* gene of *Spodoptera litura* Fabricius significantly inhibited ovarian development and decreased the egg number (Shu et al., 2011). The unique role of VgR in insect reproduction can directly affect the physiological and morphological development of insect oocytes (Han et al., 2022). This study will attempt to find answers to the following research questions. What is the effect of short-term high temperature on the reproduction of *Z. cucurbitae* (Coquillett) offsprings (F_2_ and F_3_)? Are the effects consistent with those of F_1_? How does the *VgR* gene regulate reproduction in *Z. cucurbitae* (Coquillett) after short-term high-temperature treatments? On this basis, this study first clarified the effect of different high-temperature gradient treatments for 1 h on the reproduction of *Z. cucurbitae* (Coquillett) (F_1_–F_3_) and concluded that the extreme high-temperature treatment at 45°C for 1 h stimulates the oviposition of *Z. cucurbitae* (Coquillett). *VgR* was selected as the target gene, samples with the 25°C treatment were used as the control group, and siRNA was utilized to interfere with *Z. cucurbitae* (Coquillett) with the 45°C treatment for 1 h. Our previous study showed that the *VgR* gene of *Z. cucurbitae* (Coquillett) begins to be expressed in large amounts at the age of 5 days (Lian et al., 2022). Hence, the newly emerged *Z. cucurbitae* (Coquillett) was reared at room temperature (25°C ± 1°C) after 25°C and 45°C treatment for 1 h. The expression of the *VgR* gene was interfered through the injection of siRNA at the age of 5 days to clarify the function of the *VgR* gene after short-term high-temperature treatment. The results of this study can provide theoretical and practical references for the monitoring, early warning, and management of *Z. cucurbitae* (Coquillett).

## 2 Material and methods

### 2.1 Test insect breeding management

Insect sources in this study were collected from balsam pear fields (109°29ʹ E, 19°30ʹ N) near Nada Town, Danzhou City, Hainan Province, China and then bred in the laboratory by preparing the artificial feed. Larval and adult diets are prepared according to the prescribed formula (Zeng et al., 2022). Formulas required for the preparation of feed are purchased from Hainan Qingfeng Biotechnology Co., Ltd. (China). A stable temperature-sensitive laboratory population was established. The average indoor temperature was 25°C ± 1°C, and other conditions were relative humidity of 70% ± 5% and 14 h light: 10 h dark. All newly emerged *Z. cucurbitae* (Coquillett) adults in this study were subjected to short-term high-temperature treatment in an artificial climate box (Hangzhou Qianjiang Instrument Equipment, China).

### 2.2 Setting of short-term high-temperature treatment

**FIGURE 1:**
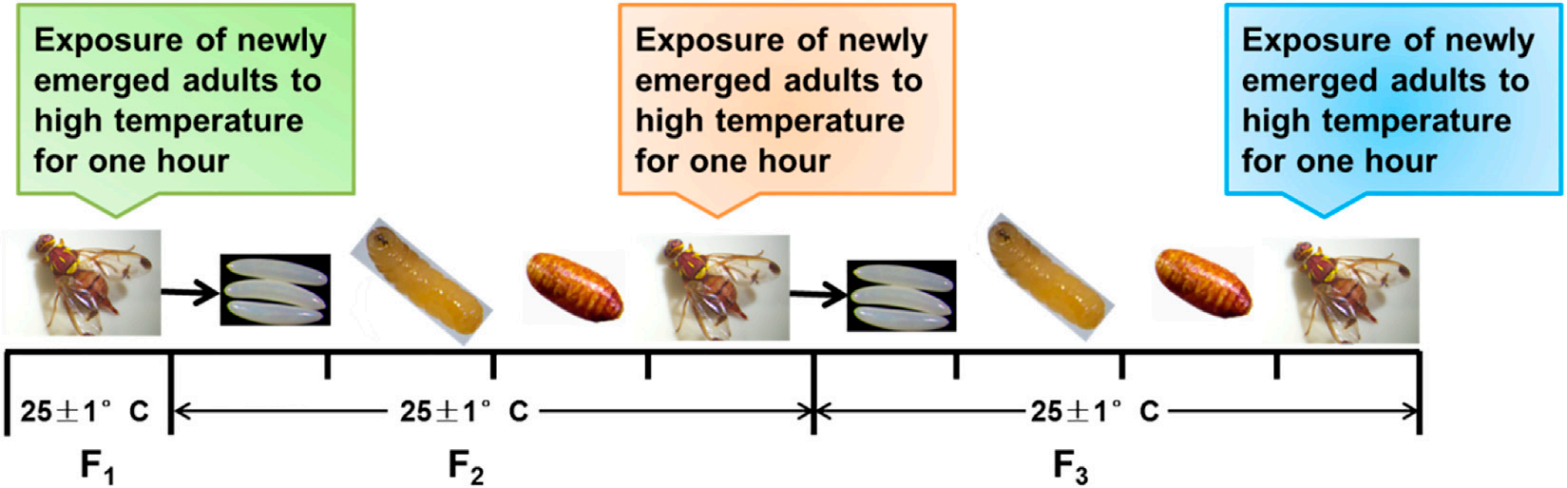
Setting of short-term high-temperature treatment. F_1_–F_3_ of *Z. cucurbitae* (Coquillett) are exposed to short-term high temperatures.

F_1_ in this study is the first generation of *Z. cucurbitae* (Coquillett), that is, contemporary *Z. cucurbitae* (Coquillett); F_2_ is the insect source of the egg laid by F_1_ to the adult; and F_3_ is the insect source of the egg laid by F_2_ to the adult. Twelve male and female newly emerged adults from each generation were released together in cages. Treatments were set to 25°C (control group), 33°C, 37°C, 41°C, and 45°C for 1 h for F_1_–F_3_ of newly emerged adults in an artificial climate chamber (Figure 1), and the 25°C treatment for 1 h was used as the control for each treatment. Indoor feeding was then implemented after treatment (25°C ± 1°C). Water, adult artificial feed, and pumpkin flakes were used for laying eggs in the cage. The egg number, oviposition days (d), and hatchability of eggs (%) were observed and recorded every day at the end of exposure to high-temperature treatments. Each cage of the 12 pairs was considered one replicate, and six replicates were established under each treatment.

### 2.3 Design and preparation of siRNA

The 21-nucleotide siRNA was designed on the basis of the conserved cDNA sequence of the *Z. cucurbitae* (Coquillett) *VgR* gene, with a positive strand of 5′- CGA​UGU​CGA​GGA​UGU​GUU​ATT-3′ and an antisense strand of 5′- UAA​CAC​AUC​CUC​GAC​AUC​GTT-3'; and the negative control (NC) is a commercially available siRNA without homology to the target cell that exerts no RNAi effect in any treatment and contains a positive strand of 5′–GGU​UCU​CCG​AAC​GUG​UCA​CGU-3′ and an antisense strand of 5′–ACG​UGA​CAC​GUU​CGG​AGA​ACC-3'. Both siRNAs were synthesized by Qingke Biotechnology Co., Ltd. (China).

### 2.4 Injection of siRNA for target genes

The siRNA dry powder (2.5 nmol) is dissolved in 125 μl of Tris-EDTA solution, and 20 μM of the siRNA solution is prepared. The newly emerged adult melon fruit fly was treated at 25°C and 45°C for 1 h in an artificial climate box. Feeding was continued at room temperature (25°C ± 1°C) after completing the treatment. Joint of the second to third abdominal segments of the abdominal backplane of the female adult was selected as the injection point and 1.25 μg (4.5 μl) of siRNA and NC were injected using the pneumatic microinjector (IM-11-2, NARISHIGE, Japan) after feeding for 5 days. The injury (using an empty injection needle in the same position) and control check (CK) were set.

### 2.5 qRT-PCR analysis of target gene expression levels

Female adults of siRNA, NC, injury, and CK groups were placed in small cages post injection. The same number of male adults were paired with female adults; given adult artificial feed, water, and pumpkin flakes for laying eggs; and reared at room temperature (25°C ± 1°C). 15 female adults were taken from siRNA, NC, injury, and CK groups at 24, 48, and 72 h postinjection. Five female adults were established as one replicate, with each group containing three replicates, snap-frozen in liquid nitrogen, and placed in an ultralow-temperature refrigerator.

The total RNA was extracted using the Animal Tissue/Cell Total RNA extraction kit (Dual Column Type) (RNAprep FastPure, Qingke Biotechnology, China). The extracted total RNA was synthesized using the reverse transcriptase kit (Goldenstar RT6 cDNA Synthesis Max, Qingke Biotechnology, China) after agarose gel electrophoresis (1.5% agarose, 1× TAE electrophoresis buffer) and absorbance detection. The cDNA was synthesized in accordance with the instructions. The cDNA product obtained *via* reverse transcription was used as the qPCR template after three rounds of dilution. The size of the band obtained through agarose gel electrophoresis was consistent with the expected size, and stray bands were absent. The fusion curve of quantitative real-time PCR presented only one signal peak, thereby indicating the high specificity of primers and reliable results. *Succinate dehydrogenase flavoprotein* gene was selected as the housekeeping gene on the basis of previous screening (Zhou, 2016). Primers used in this experiment are listed in Table 1. The reaction system contained 1.0 μl of cDNA template, 10.0 μl of 2×T5 Fast qPCR Mix (SYBR Green I), 1 μl of the upstream primer, 1 μl of the downstream primer, and 7.0 μl of ddH_2_O. The reaction procedure is presented as follows: predenaturation at 95°C for 2 min, denaturation at 95°C for 15 s, annealing at 60°C for 15 s, and extension at 72°C for 20 s. A total of 41 cycles were carried out with three replicates per sample. Relative quantitative analysis mode was selected and original data were derived after the reaction. *C*_T_ values of the internal reference gene and the gene to be examined were determined using the 2^−∆∆CT^ method (Livak and Schmittgen, 2001). Multiple relationships of differential expression patterns of samples were obtained.

**TABLE 1:** Primer sequence information of target gene and reference gene.

Primer name	Primer sequences (5′-3′)	Use of primers
*Vitellogenin receptor*-F	TGC​TTT​CCC​GGT​TAT​CGC​TT	Target gene amplification in qPCR
*Vitellogenin receptor-*R	AAC​GTA​ATC​GGT​TGC​TCC​GT	
*Vitellogenin-1*-F	TGC​CAC​GTG​ACT​TAA​TCG​GT	
*Vitellogenin-1-*R	ATG​CTG​TGG​CTA​GAG​GCC​AT	
*Vitellogenin-2*-F	GCT​TGT​AAT​GAG​TCG​GTG​GCG	
*Vitellogenin-2-*R	AGT​GGC​GCA​AAG​AAA​TGC​CT	
*Vitellogenin-3*-F	TTT​GCT​CCT​CAG​CAC​TCT​CA	
*Vitellogenin-3-*R	GCG​CCA​TAT​TTG​ATC​GGC​A	
*succinate dehydrogenase flavoprotein*-F	TTG​ATT​TCA​AAA​TAG​GCG​CAG​TG	References gene amplification in qPCR
*succinate dehydrogenase flavoprotein*-R	CGA​TGG​TAC​ACG​CAT​AAG​GC	

### 2.6 Effect of interfering with target genes on oviposition of *Z. cucurbitae* (Coquillett)

Five female adults from siRNA, NC, injury, and CK groups were placed in small cages and five male adults were paired as one replicate postinjection. Six replicates were established; given an adult artificial feed, water, and pumpkin flakes for laying eggs; and reared at room temperature (25°C ± 1°C). The egg number, oviposition days (d), and hatchability of eggs (%) were recorded.

### 2.7 Effect of interfering with target genes on ovarian development of *Z. cucurbitae* (Coquillett)

Female adults of siRNA, NC, injury, and CK groups were placed in small cages postinjection. The same number of male adults were paired with female adults; given an adult artificial feed, water, and pumpkin flakes for laying eggs; and reared at room temperature (25°C ± 1°C). The 11-, 12-, and 13-day-old female adults were dissected at 5 days postinjection, with five female adults dissected per treatment per day and their ovaries photographed and recorded.

### 2.8 Statistical analysis

Completely randomized ANOVA and Tukey’s multiple comparisons were performed on the data using Excel (version 2021) and SPSS (version 26.0). Proportional data were first transformed using the square root of the inverse sine and then analyzed *via* ANOVA. All values in the results are presented as the average number ±standard error.

## 3 Results

### 3.1 Effect of short-term high temperature on the reproduction of *Z. cucurbitae* (Coquillett)

**FIGURE 2:**
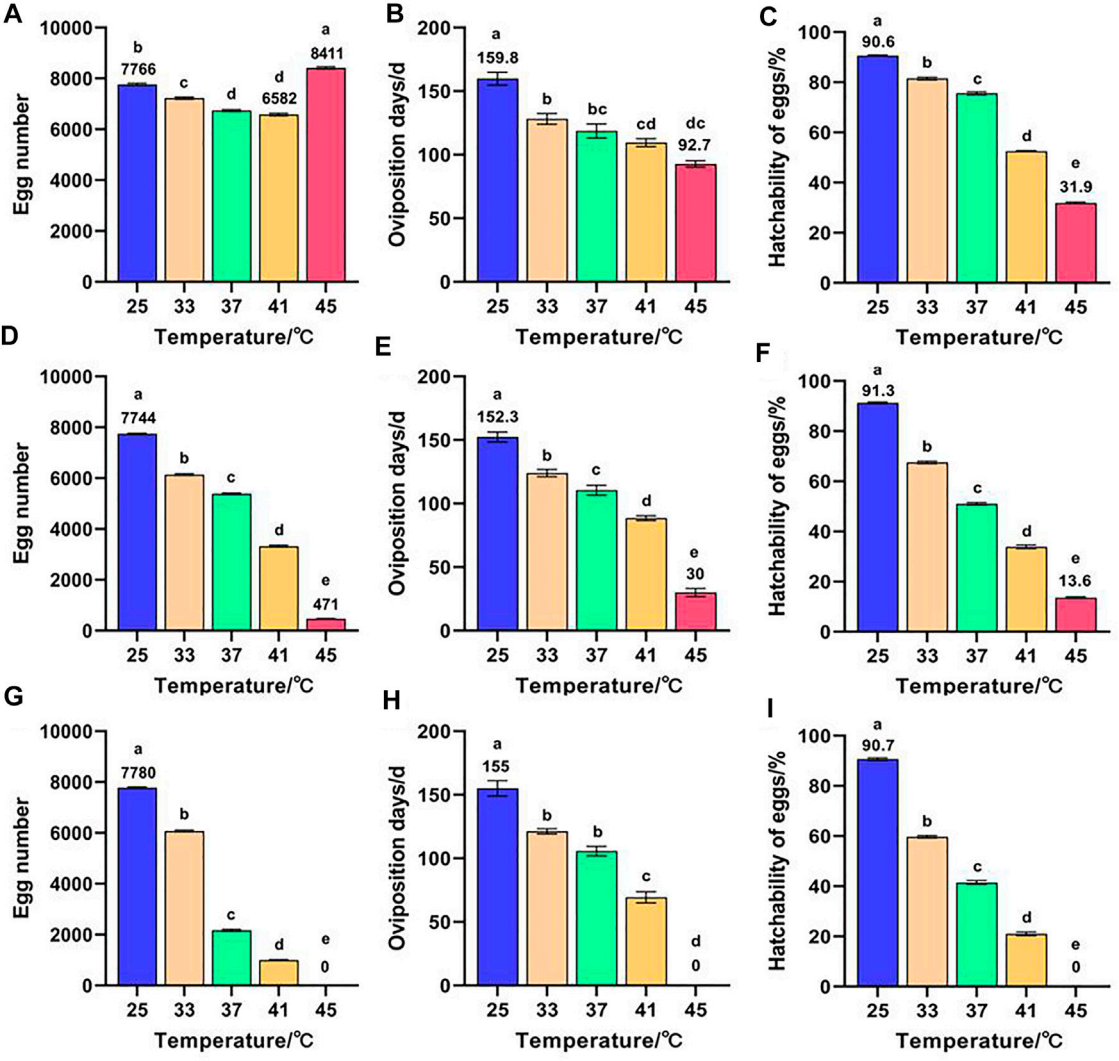
Effects of short-term high temperature on the reproduction of *Z. cucurbitae* (Coquillett). **(A)** Egg number of F_1_ (*F* = 268.599, *p* = 0.0001). **(B)** Oviposition days of F_1_ (*F* = 34.15, *p* = 0.0001). **(C)** Hatchability of eggs of F_1_ (*F* = 3,456.539, *p* = 0.0001). **(D)** Egg number of F_2_ (*F* = 8,460.934, *p* = 0.0001). **(E)** Oviposition days of F_2_ (*F* = 200.296, *p* = 0.0001). **(F)** Hatchability of eggs of F_2_ (*F* = 3,305.611, *p* = 0.0001). **(G)** Egg number of F_3_ (*F* = 12,589.238, *p* = 0.0001). **(H)** Oviposition days of F_3_ (*F* = 228.794, *p* = 0.0001). **(I)** Hatchability of eggs of F_3_ (*F* = 4,570.108, *p* = 0.0001). All values in the figure are presented as mean ± standard error. Letters above the bars indicate significant differences (*p* ≤ 0.05).

Effects of short-term high temperature on the reproduction of *Z. cucurbitae* (Coquillett) are shown in Figure 2. The egg number of F_1_ was the highest in the 45°C treatment and lowest in the 41°C treatment with 8,411 and 6,582 eggs, respectively. This finding is significantly different from that of the control group (7,766 eggs) (Figure 2A). Both oviposition days (Figure 2B) and hatchability of eggs (Figure 2C) of F_1_ decreased with the increase of temperature and reached the minimum in the 45°C treatment at 92.7 days and 31.9%. This finding is significantly lower than that of the control group at 159.8 days and 90.6%. The egg number (Figure 2D), oviposition days (Figure 2E), and hatchability of eggs (Figure 2F) in F_2_ all decreased with the increase of temperature and reached the minimum in the 45°C treatment at 471 eggs, 30 days, and 13.6%. This finding is significantly lower than that of the control group (7,744 eggs, 152.3 days, and 91.3%). The egg number (Figure 2G), oviposition days (Figure 2H), and hatchability of eggs (Figure 2I) of the control group in F_3_ were significantly higher than those of the high-temperature treated group. Moreover, all F_3_ died when offsprings (F_2_ and F_3_) were continuously exposed to short-term high temperature at 45°C for 1 h. Therefore, the short-term high temperature, except for F_1_ treated at 45°C for 1 h to stimulate oviposition, was generally unfavorable to the reproduction of *Z. cucurbitae* (Coquillett).

### 3.2 qRT-PCR analysis of target gene expression levels

#### 3.2.1 Effect of interfering with target genes on the expression of the *VgR* gene in *Z. cucurbitae* (Coquillett)

**FIGURE 3:**
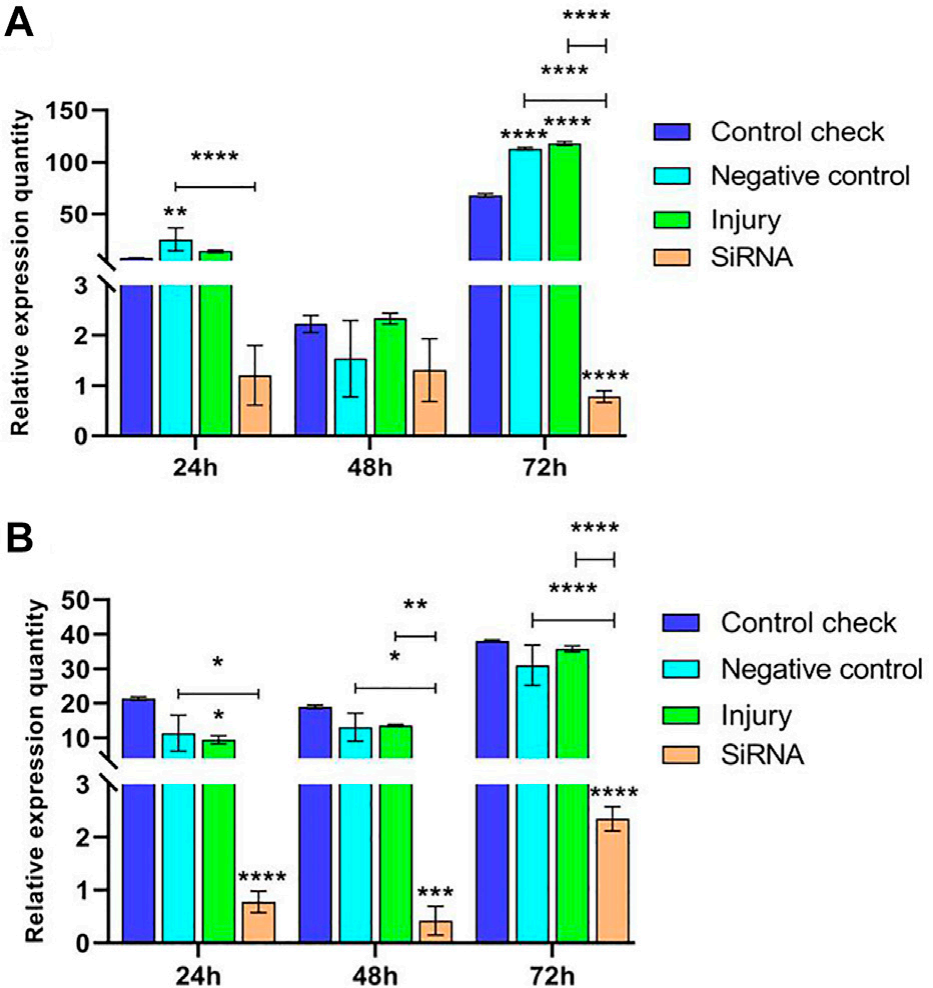
Effects of interfering target genes on the *Vitellogenin receptor* gene expression in *Z. cucurbitae* (Coquillett). **(A)** Interfering target genes after the 25°C treatment for 1 h (*F*_(3, 24)_ = 119.9, *p* < 0.0001; *F*_(2, 24)_ = 557.3, *p* < 0.0001; *F*_(6, 24)_ = 75.67, *p* < 0.0001). **(B)** Interfering target genes after the 45°C treatment for 1 h (*F*_(3, 24)_ = 49.97, *p* < 0.0001; *F*_(2, 24)_ = 49.26, *p* < 0.0001*; F*_(6, 24)_ = 4.778, *p* = 0.0025). All values in the figure are presented as mean ± standard error. *, **, ***, and **** above the histograms indicate the values that differ significantly between treatments *p* ≤ 0.05, *p* ≤ 0.01, *p* ≤ 0.001, and *p* ≤ 0.0001, respectively.

Expression levels of the *VgR* gene were investigated by injecting siRNA into 5-day-old female adults and collecting samples after 24, 48, and 72 h. The results showed that the relative expression of CK, NC, and injury groups all peaked at 72 h in the 25°C treatment and the siRNA group constantly maintained a low level of expression in the three time periods. The relative expression of NC and injury groups was significantly higher than that of the siRNA group at 24 h, and the difference among the groups at 48 h was insignificant. The relative expression of CK, NC, and injury groups was significantly higher than that of the siRNA group at 72 h (Figure 3A). This finding indicated that the expression of the *VgR* gene is effectively interfered. The relative expression of each group in the 45°C treatment showed an increasing trend and reached the peak at 72 h. The relative expression of the siRNA group showed a lower level of expression in the three time periods; and the relative expression of CK, NC, and injury groups was significantly higher than that of the siRNA group in the three time periods (Figure 3B). The maximum relative expression of CK, NC, and injury groups in the 45°C treatment was higher than that in the 25°C treatment.

#### 3.2.2 Effect of interfering with target genes on the expression of *Vg-1* gene in *Z. cucurbitae* (Coquillett)

**FIGURE 4:**
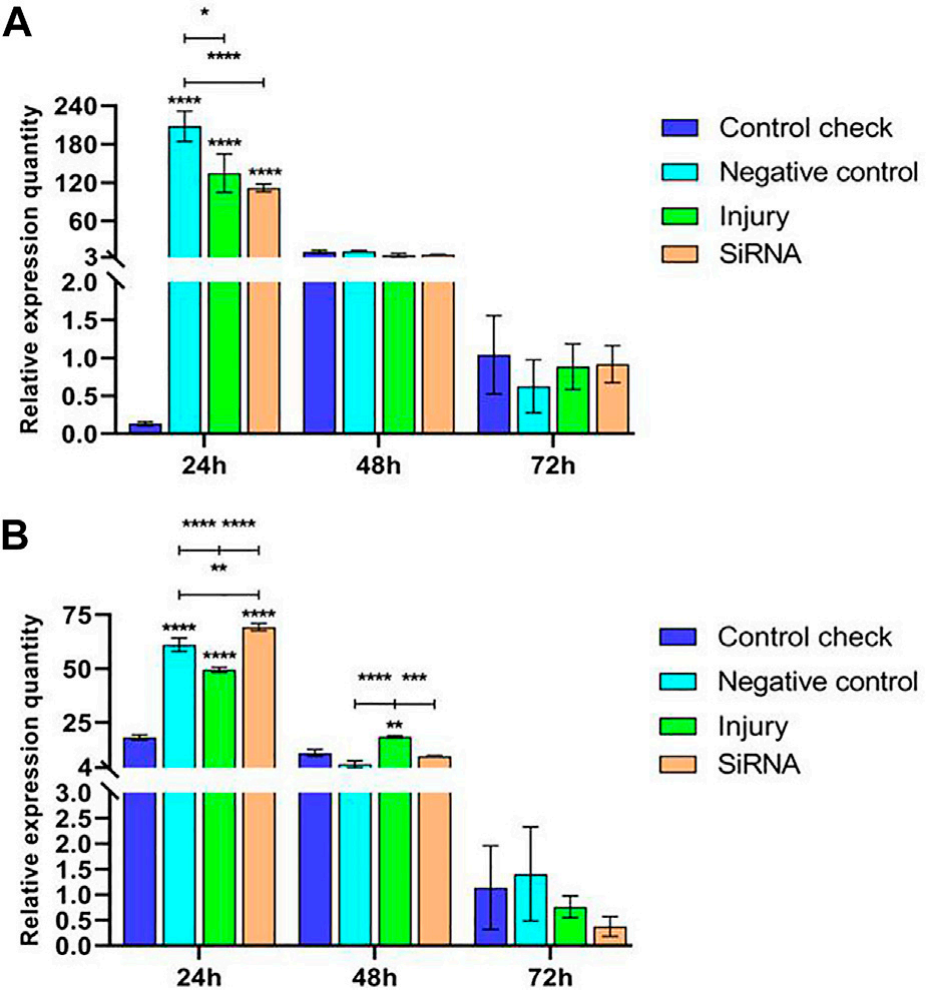
Effects of interfering target genes on *Vitellogenin-1* gene expression in *Z. cucurbitae* (Coquillett). **(A)** Interfering target genes after the 25°C treatment for 1 h (*F*_(3, 24)_ = 19.43, *p* < 0.0001; *F*_(2, 24)_ = 124.6, *p* < 0.0001; *F*_(6, 24)_ = 19.61, *p* < 0.0001). **(B)** Interfering target genes after the 45°C treatment for 1 h (*F*_(3, 24)_ = 84.23, *p* < 0.0001; *F*_(2, 24)_ = 1,436, *p* < 0.0001; *F*_(6, 24)_ = 103.4, *p* < 0.0001). All values in the figure are presented as mean ± standard error. *, **, ***, and **** above the histograms indicate the values that differ significantly between treatments *p* ≤ 0.05, *p* ≤ 0.01, *p* ≤ 0.001, and *p* ≤ 0.0001, respectively.

Expression levels of the *Vg-1* gene were explored by injecting siRNA into 5-day-old female adults and then collecting samples after 24, 48, and 72 h. The results showed that relative expressions of NC, injury, and siRNA groups all decrease after reaching the maximum at 24 h in the 25°C treatment. CK was expressed at lower levels in all three time periods, significantly lower than other groups at 24 h, and not significantly different from other groups at 48 and 72 h (Figure 4A). The expression of the siRNA was significantly higher than that of other groups at 24 h and not significantly different from that of other groups at 72 h in the 45°C treatment. The relative expression of all groups showed a decreasing trend and peaked at 24 h, and the maximum relative expression of NC, injury, and siRNA in the 45°C treatment was lower than that in the 25°C treatment (Figure 4B).

#### 3.2.3 Effect of interfering with target genes on the expression of *Vg-2* gene in *Z. cucurbitae* (Coquillett)

**FIGURE 5:**
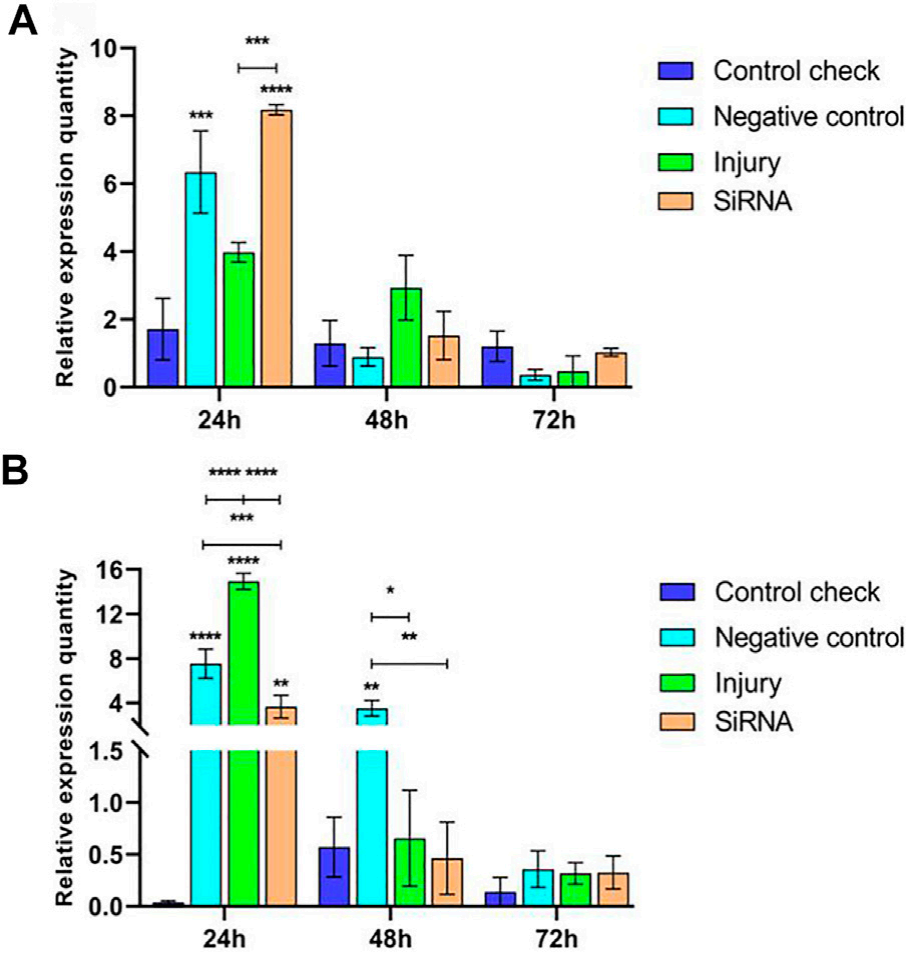
Effects of interfering target genes on *Vitellogenin-2* gene expression in *Z. cucurbitae* (Coquillett). **(A)** Interfering target genes after the 25°C treatment for 1 h (*F*_(3, 24)_ = 5.937, *p* = 0.0035; *F*_(2, 24)_ = 51.31, *p* < 0.0001; *F*_(6, 24)_ = 8.164, *p* < 0.0001). **(B)** Interfering target genes after the 45°C treatment for 1 h (*F*_(3, 24)_ = 43.28, *p* < 0.0001; *F*_(2, 24)_ = 126.1, *p* < 0.0001; *F*_(6, 24)_ = 38.32, *p* < 0.0001). All values in the figure are presented as mean ± standard error. *, **, ***, and **** above the histograms indicate the values that differ significantly between treatments *p* ≤ 0.05, *p* ≤ 0.01, *p* ≤ 0.001, and *p* ≤ 0.0001, respectively.

Expression levels of the *Vg-2* gene were analyzed by injecting siRNA into 5-day-old female adults and collecting samples after 24, 48, and 72 h. The results showed that relative expressions of NC, injury, and siRNA groups all decrease after reaching the maximum at 24 h in the 25°C treatment. Relative expressions of CK showed stable changes in the three time periods without significant differences. Relative expressions of the siRNA group were significantly higher than those of the CK and injury groups at 24 h and not significantly different from other groups at 48 and 72 h (Figure 5A). Relative expressions of NC, injury, and siRNA groups all decrease after reaching the maximum at 24 h in the 45°C treatment. The CK showed low levels of expression in the three time periods; relative expressions of the siRNA group were significantly lower than those of NC and injury groups at 24 h, significantly lower than those of NC at 48 h, and not significantly different from other groups at 72 h (Figure 5B).

#### 3.2.4 Effect of interfering with target genes on the expression of *Vg-3* gene in *Z. cucurbitae* (Coquillett)

**FIGURE 6:**
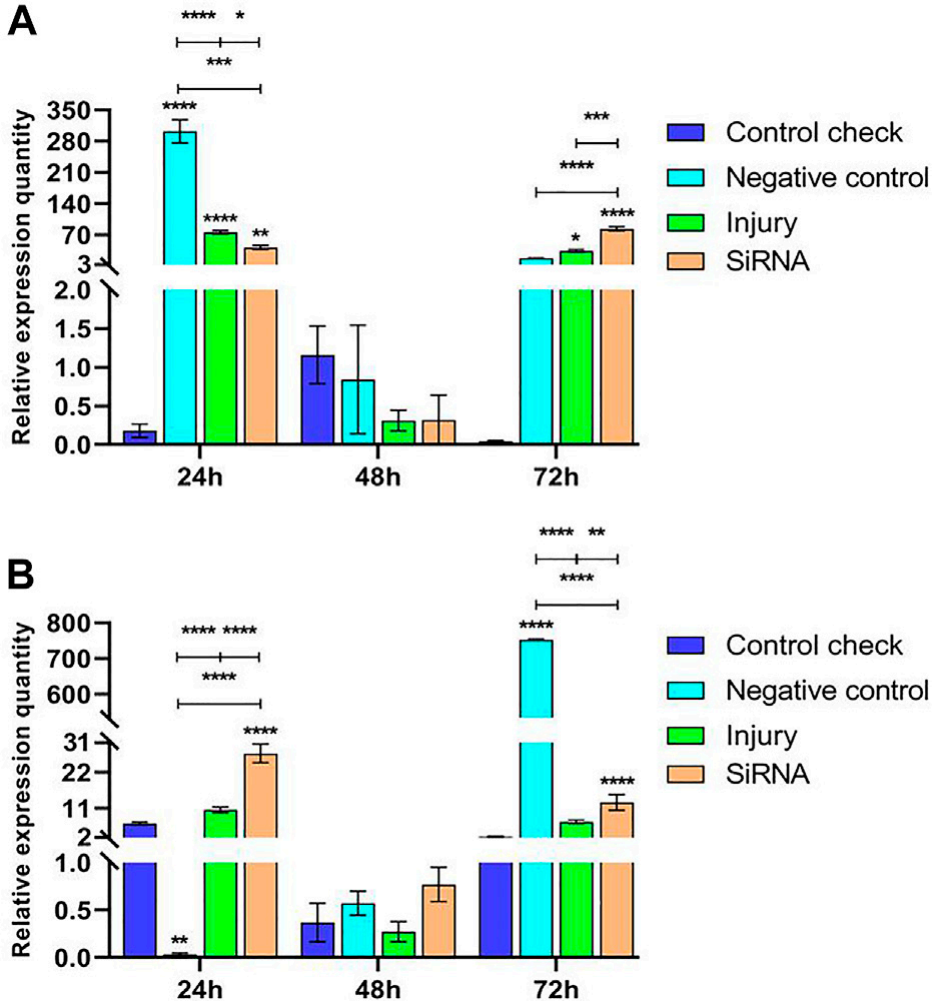
Effects of interfering target genes on *Vitellogenin-3* gene expression in *Z. cucurbitae* (Coquillett). **(A)** Interfering target genes after the 25°C treatment for 1 h (*F*_(3, 24)_ = 95.43, *p* < 0.0001; *F*_(2, 24)_ = 184.1, *p* < 0.0001; *F*_(6, 24)_ = 110.9, *p* < 0.0001). **(B)** Interfering target genes after the 45°C treatment for 1 h (*F*_(3, 24)_ = 30,361 *p* < 0.0001; *F*_(2, 24)_ = 32,129, *p* < 0.0001; *F*_(6, 24)_ = 32,203, *p* < 0.0001). All values in the figure are presented as mean ± standard error. *, **, ***, and **** above the histograms indicate the values that differ significantly between treatments *p* ≤ 0.05, *p* ≤ 0.01, *p* ≤ 0.001, and *p* ≤ 0.0001, respectively.

Expression levels of the *Vg-3* gene were examined by injecting siRNA into 5-day-old female adults and collecting samples after 24, 48, and 72 h. The results showed that relative expressions of NC, injury, and siRNA groups all downregulate and then upregulate in the 25°C treatment. The CK showed a low level of expression in the three time periods; the relative expression of the siRNA group was significantly lower than that of NC and injury groups at 24 h, not significantly different from that of other groups at 48 h, and significantly higher than that of other groups at 72 h (Figure 6A). Relative expressions of CK, injury, and siRNA groups first downregulated and then upregulated in the 45°C treatment. Relative expressions of the siRNA group at 24 h were significantly higher than that of other groups, and the relative expression of NC continuously upregulated and reached the peak at 72 h. The maximum relative expression was significantly higher than that of other groups (Figure 6B).

### 3.3 Effect of interfering with target genes on oviposition of *Z. cucurbitae* (coquillett)

**FIGURE 7:**
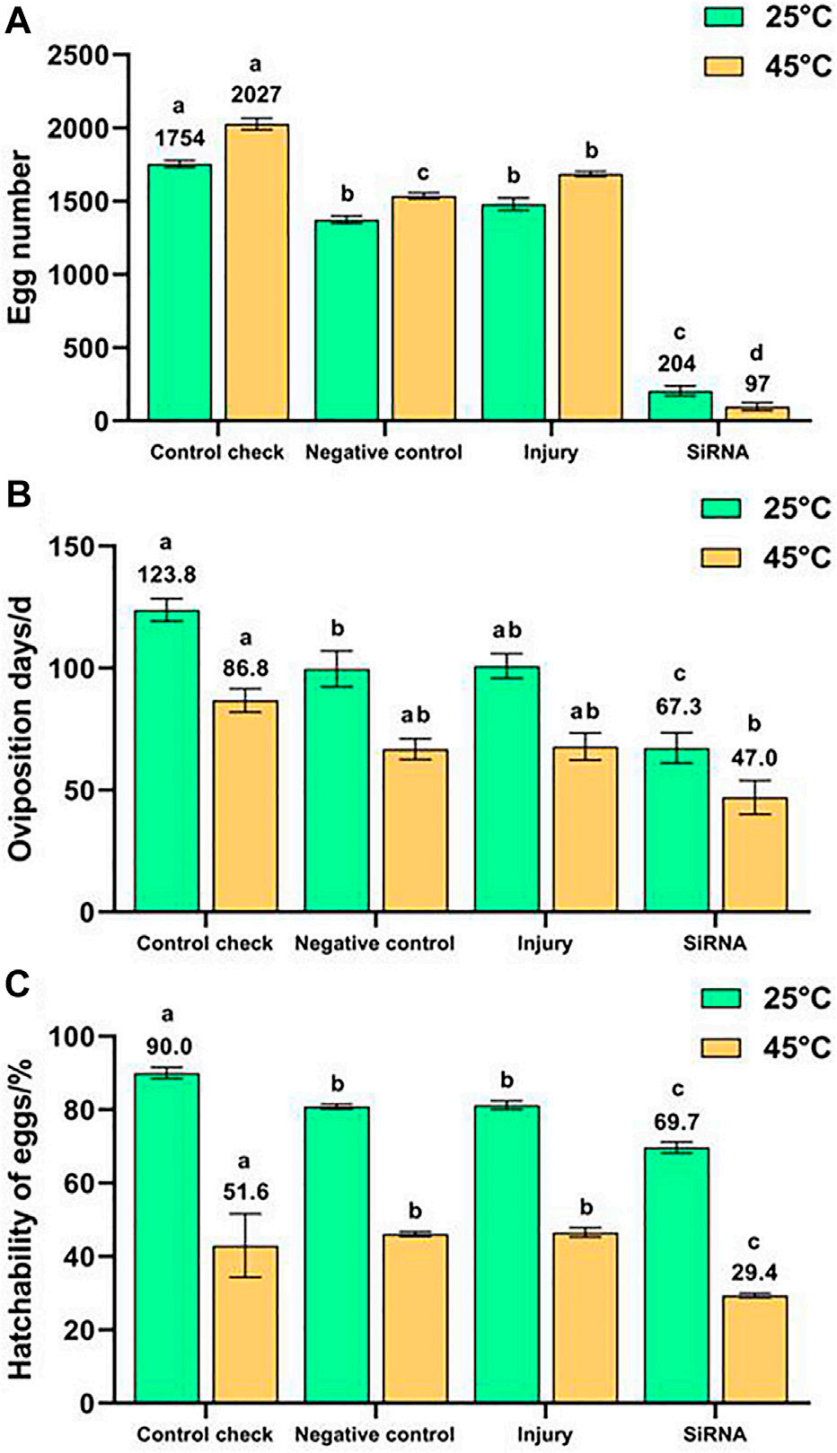
Effects of interfering target genes on oviposition of *Z. cucurbitae* (Coquillett). **(A)** Egg number (25°C, *F* = 447.038, *p* = 0.0001; 45°C, *F* = 1,084.647, *p* = 0.0001). **(B)** Oviposition days (25°C, *F* = 15.341, *p* = 0.0001; 45°C, *F* = 8.163, *p* = 0.0011). **(C)** Hatchability of eggs (25°C, *F* = 42.001, *p* = 0.0001; 45°C, *F* = 123.341, *p* = 0.0001). All values in the figure are presented as mean ± standard error. The letters above the bars indicate the significant difference (*p* ≤ 0.05).

The 5-day-old female adults were injected with siRNA, and their egg number (Figure 7A), oviposition days (Figure 7B), and hatchability of eggs (Figure 7C) were recorded. The egg number, oviposition days, and hatchability of eggs of the siRNA group were the lowest with 204 eggs, 67.3 days, and 69.7%, which were significantly lower than those of other groups and lower than those of the CK (1754 eggs, 123.8 days, and 90.0%, respectively) by about 88.4%, 45.6%, and 22.6%, respectively, in the 25°C treatment. The egg number, oviposition days, and hatchability of eggs of the siRNA group were the lowest with 97 eggs, 47.0 days, and 29.4%, which were significantly lower than those of the CK (2027 eggs, 86.8 days, and 51.6%, respectively) by about 95.2%, 45.9% and 43.0%, respectively, in the 45°C treatment. The egg number of CK, NC, and injury groups were all higher than those at 25°C for 1 h after the 45°C treatment for 1 h. By contrast, the siRNA group demonstrated a lower egg number at 45°C than that at 25°C. In conclusion, silencing the *VgR* gene effectively inhibited the reproduction of *Z. cucurbitae* (Coquillett) and made the 45°C treatment for 1 h no longer stimulating the oviposition of *Z. cucurbitae* (Coquillett).

### 3.4 Effect of interfering with target genes on ovarian development of *Z. cucurbitae* (Coquillett)

**FIGURE 8:**
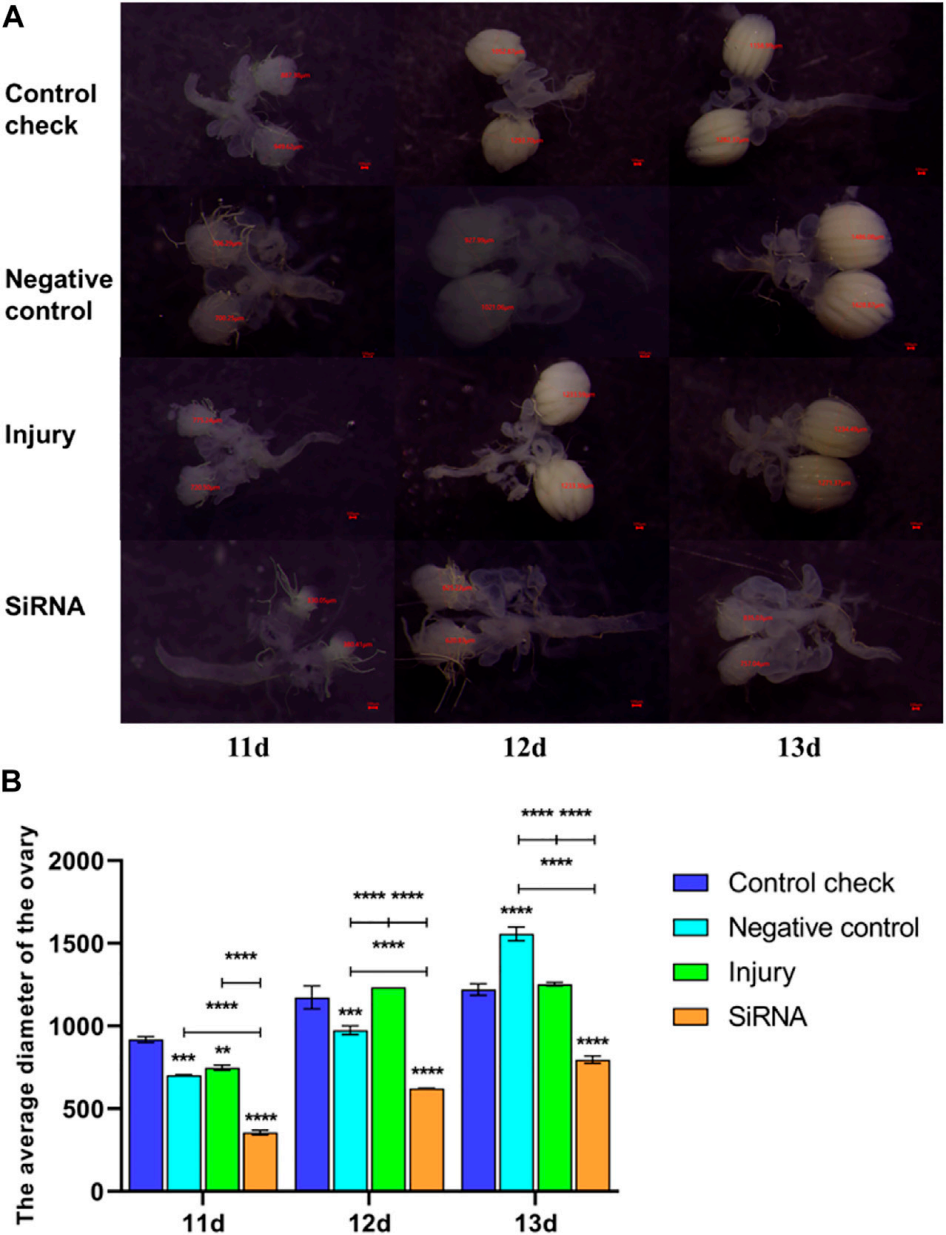
Effects of interfering target genes on ovarian development of *Z. cucurbitae* (Coquillett) after the 25°C treatment for 1 h. **(A)** Rate of ovarian development. The red line denotes the diameter of the ovary (μm). **(B)** Average diameter of the ovary (*F*_(3, 24)_ = 223.1, *p* < 0.0001; *F*_(2, 24)_ = 339.6, *p* < 0.0001; *F*_(6, 24)_ = 27.24, *p* < 0.0001). All values in the figure are presented as mean ± standard error. *, **, ***, and **** above the histograms indicate the values that differ significantly between treatments *p* ≤ 0.05, *p* ≤ 0.01, *p* ≤ 0.001, and *p* ≤ 0.0001, respectively.

**FIGURE 9:**
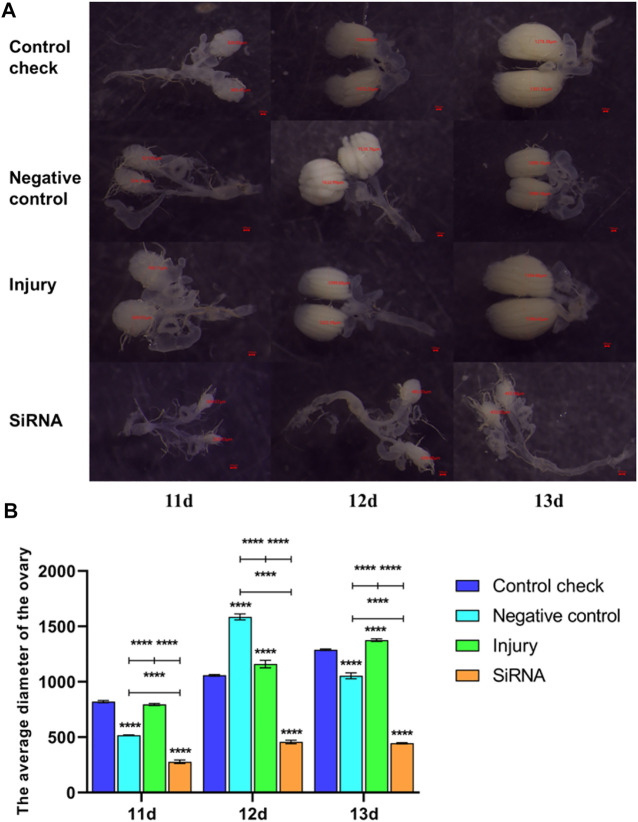
Effects of interfering target genes on ovarian development of *Z. cucurbitae* (Coquillett) after the 45°C treatment for 1 h. **(A)** Rate of ovarian development. The red line denotes the diameter of the ovary (μm). **(B)** Average diameter of the ovary (*F*_(3, 24)_ = 1,158, *p* < 0.0001; *F*_(2, 24)_ = 901.2, *p* < 0.0001; *F*_(6, 24)_ = 183.0, *p* < 0.0001). All values in the figure are presented as mean ± standard error. *, **, ***, and **** above the histograms indicate the values that differ significantly between treatments *p* ≤ 0.05, *p* ≤ 0.01, *p* ≤ 0.001, and *p* ≤ 0.0001, respectively.

The 5-day-old female adults were injected with siRNA, and the 11-, 12-, and 13-day-old female adults were dissected 5 days postinjection. The siRNA group demonstrated significantly smaller ovary diameters and significantly slower ovary development rate than other groups in the 25°C (Figure 8) and 45°C (Figure 9) treatments. In conclusion, silencing the *VgR* gene decelerated the development of ovaries in *Z. cucurbitae* (Coquillett), and the ovary diameter of the siRNA group in the 45°C treatment was smaller than that in the 25°C treatment in all three time periods. This finding indicated that the extremely high temperature of 45°C for 1 h inhibits the ovary development of *Z. cucurbitae* (Coquillett).

## 4 Discussion

Short-term high-temperature can exert a remarkable impact on pest reproduction because pests usually occur for several generations in a year (Raza et al., 2015). Short-term high-temperature treatments, except for F_1_ treated at 45°C for 1 h to stimulate oviposition, presented a general adverse effect on the reproduction of *Z. cucurbitae* (Coquillett), and fecundity of *Z. cucurbitae* (Coquillett) decreased continuously with the increase of treatment temperature in this study. Compared with the control (25°C), the egg number of *Bactrocera dorsalis* (Hendel) female adults reduced after the 35°C treatment for 2 h. However, the egg number of *B. dorsalis* (Hendel) after the 40°C treatment for 2 h was 1,445.4 eggs/d, which is significantly higher than that of the control (691.89 eggs/d) (Ren et al., 2010). The exposure of female *Grapholitha molesta* to the 38°C treatment for 4 h resulted in a fecundity increase of 8.9% but a significant decrease in the hatchability of eggs (Liang et al., 2014). These findings are consistent with those of our research results. Studies have shown that insects stimulated by certain environmental conditions, such as high temperatures, produce a “toxic excitatory effect.” This effect is a specific temperature-adapted survival coping strategy and mechanism against heat stress (Villalpando et al., 2008; Hoffmann and Sgrò, 2011). The egg number of F_2_ and F_3_ after exposure to 45°C for 1 h was much lower than that of the control group, thereby indicating that the “toxic excitatory effect” is unstable and difficult to reproduce between generations. Hence, the “toxic excitatory effect” was affected not only by the time of high-temperature treatment but also the number of high-temperature treatments. The egg number, oviposition days, and hatchability of eggs in the high-temperature treatment group showed a downward trend with the increase of generations; this finding is consistent with that of high-temperature stress on red *Acyrthosiphon pisum* Harris that led to a significant decrease in the fertility of its progeny (Sun and Liu, 2016). The death of F_3_ after the 45°C treatment for 1 h indicated that the negative influence of high temperature on maternal generation is passed on to the offspring and heat resistance of the offspring is reduced. Therefore, high-temperature characteristic factors, such as intensity, duration, and frequency in the field should be fully considered when preventing and controlling *Z. cucurbitae* (Coquillett) during high-temperature seasons, and short-term high-temperature characteristic factors can also be used to prevent and control *Z. cucurbitae* (Coquillett).

VgR is a specific receptor for Vg that ensures insects receive sufficient nutrients during vitellogenesis and plays a vital role in the maturation of insect ovaries (Lin et al., 2013). Interfering with the *VgR* gene expression in *Cadra cautella* using dsRNA significantly reduced the fertility and hatchability of eggs in female adults (Husain et al., 2020). Interference with the *NlVgR* gene of *Nilaparvata lugens* (Stål) resulted in the inhibition of ovarian development (Lu et al., 2015). Silencing of the *HaVGR* gene of *Helicoverpa armigera* (Hübner) led to immature ovary development and significantly reduced egg number (Zhang et al., 2016). The injection of siRNA in our study resulted in the low expression of the *VgR* gene at 24, 48, and 72 h and significant downregulation compared with CK, NC, and injury groups at 72 h. The egg number, oviposition days, and hatchability of eggs were significantly lower than those of other groups after interference. The dissection of ovaries revealed that the ovary development rate and diameter are also significantly lower than those of other groups after interference. This finding indicated that the *VgR* gene is involved in the reproductive regulation of *Z. cucurbitae* (Coquillett) and VgR is essential in the reproduction of *Z. cucurbitae* (Coquillett).

The expression of the *VgR* gene in CK, NC, and injury groups after the 45°C treatment for 1 h was lower than that after the 25°C treatment for 72 h. This finding demonstrated that the short-term high temperature downregulates the *VgR* gene expression. *Ostrinia furnacalis* exposed to ultraviolet (UV-A) for 0.5, 1, and 1.5 h resulted in the significantly lower expression of the *VgR* gene than that of the control (Liu et al., 2021). *O. furnacalis* consumes VgR when exposed to UV-A stress to maintain the strength of its defense system as well as resist, reduce, or avoid the damage caused by UV-A stress (Meng et al., 2009). The consumption of VgR by *Z. cucurbitae* (Coquillett) in this work for obtaining sufficient energy and resisting high-temperature stress led to the downregulation of *VgR* gene expression. The egg number of CK, NC, and injury groups with the 45°C treatment for 1 h was higher than that with the 25°C treatment for 1 h. This finding further demonstrated the “toxic excitatory effect” and oviposition stimulation in *Z. cucurbitae* (Coquillett) under the 45°C treatment for 1 h, except for the siRNA group. Hence, silencing the *VgR* gene will eliminate the “toxic excitation effect.” The egg number was significantly lower in both the NC and injury groups than that in the CK likely because the wound caused by NC and injury will lead to the partial consumption of VgR by *Z. cucurbitae* (Coquillett) to repair it. Hence, the egg number is reduced because the nutrients required for egg development decreased.

Vg is a precursor reserve protein of yolk protein, which is taken up and accumulated by oocytes during VgR-mediated endocytosis to provide the embryo with required nutrients (Piulachs et al., 2003). The siRNA injection in our study increased the expression level of *Vg-1*, *Vg-2*, and *Vg-3* genes in the siRNA group compared with that in the CK because it downregulated the *VgR* gene expression and reduced the combination of Vg and VgR. The expression of the *BdVgR* gene significantly downregulated, the expression of its ligand *BdVp1* gene significantly upregulated at 72 h after injection, and the ovarian development remained unchanged when dsRNA was used to silence the *BdVgR* gene of *B. dorsalis* (Hendel) (Cong et al., 2015). This result is basically consistent with those of our experiment. The expression of the *Vg* gene in NC, injury, and siRNA groups at 24 h was significantly higher than that in the CK at 25°C; thus, the damage caused by injection to *Z. cucurbitae* (Coquillett) may upregulate the *Vg* gene expression, and *O. furnacalis* exposed to UV-A for 3.5 h revealed the rapid upregulation of the *Vg* gene expression (Liu et al., 2020). Studies have shown that Vg is a multifunctional protein (Awde et al., 2020) that can provide nutrients required in egg development and improve the insect’s antioxidant and stress resistance (Guo, 2010). Damage may stimulate *Z. cucurbitae* (Coquillett) to secrete Vg as a way to improve its resistance to damage and thus increase the expression of the *Vg* gene.

In conclusion, this study combined apparent biological and molecular biology to investigate the effects of short-term high-temperature treatments on the reproduction of *Z. cucurbitae* (Coquillett). The following conclusions can be drawn from this study. Short-term high-temperature treatments, except for the F_1_ treatment at 45°C for 1 h to stimulate the oviposition, were generally unfavorable to the reproduction of *Z. cucurbitae* (Coquillett). All F_3_ died when offsprings (F_2_ and F_3_) were continuously exposed to the short-term high-temperature treatment of 45°C for 1 h. The *VgR* gene was involved in the reproductive regulation of *Z. cucurbitae* (Coquillett), and VgR was essential in the reproduction of *Z. cucurbitae* (Coquillett) after short-term high-temperature treatments. Vg was as important as VgR because Vg is the main source of embryos. Therefore, the function of the Vg gene will be explored using RNAi or CRISPR-Cas9 gene editing techniques and the Vg gene will be combined with short-term high temperatures in future investigations to examine reproduction regulation in *Z. cucurbitae* (Coquillett) after short-term high-temperature treatments.

## Data Availability

The original contributions presented in the study are included in the article/supplementary material, further inquiries can be directed to the corresponding author.
